# Commentary: “An Evaluation of Universal Grammar and the Phonological Mind”—UG Is Still a Viable Hypothesis

**DOI:** 10.3389/fpsyg.2016.01029

**Published:** 2016-07-14

**Authors:** Iris Berent

**Affiliations:** Phonology and Reading Laboratory, Department of Psychology, Northeastern University, BostonMA, USA

**Keywords:** phonology, universal grammar, core knoweldge, innateness, sonority

## Abstract

Everett (2016b) criticizes *The Phonological Mind* thesis (Berent, 2013a,b) on logical, methodological and empirical grounds. Most of Everett’s concerns are directed toward the hypothesis that the phonological grammar is constrained by universal grammatical (UG) principles. Contrary to Everett’s logical challenges, here I show that the UG hypothesis is readily falsifiable, that universality is not inconsistent with innateness (Everett’s arguments to the contrary are rooted in a basic confusion of the UG phenotype and the genotype), and that its empirical evaluation does not require a full evolutionary account of language. A detailed analysis of one case study, the syllable hierarchy, presents a specific demonstration that people have knowledge of putatively universal principles that are unattested in their language and these principles are most likely linguistic in nature. Whether Universal Grammar exists remains unknown, but Everett’s arguments hardly undermine the viability of this hypothesis.

*The Phonological Mind* thesis (Berent, 2013a,b), according to Everett (2016b), is plainly silly. It defends a formal linguistic account that is blatantly false, its experimental support is confounded by trivial “perceptual” factors, and its biological bases are at best tenuous. The problem, according to Everett, is not limited to this particular theory of phonology, or even language. Rather, it is an entire class of nativist accounts of cognition, specifically, the “core knowledge” hypothesis, that is bankrupt. *The Phonological Mind* thesis, then, exemplifies what has gone so wrong in cognitive nativism.

I believe Everett’s concerns are unfounded. Everett’s broader objections to cognitive nativism are rooted in an erroneous conflation of explanations at the cognitive and biological levels, an implausible view of the links between the cognitive phenotype and the genotype, and a disregard for a large experimental literature that counters his assertions. Similarly, in his attack on *The Phonological Mind’s* thesis, Everett ignores most of the relevant experimental evidence, he systematically misrepresents my claims, and his characterization of my approach is disingenuous. Whether Universal Grammar exists remains unknown—a fact that (contrary to Everett’s claims) I openly acknowledge. But Everett’s superficial reading of the scientific literature and his incendiary rhetoric do not challenge this hypothesis.

My reply proceeds as follows. I first consider Everett’s broader critique of cognitive nativism and address his logical and methodological concerns about the falsification of nativist claims and their congruency with genetic and evolutionary evidence. Having established some ground rules for testing the core knowledge hypothesis, I proceed to consider the evidence for innate phonological constraints by evaluating the empirical evidence for UG in a particular case study—the restrictions on syllable structure. Everett’s remaining arguments against *The Phonological Mind* thesis are discussed in the final section. While my reply focuses mostly on the arguments in Everett (2016a), the broader context is set in reference to his upcoming book, *Dark Matter of the Mind*, of which Everett (2016b) is an excerpt.

## Evaluating Cognitive Nativism

To appreciate Everett’s (2016b) critique of nativist theories of language, it is important to consider it within his broader critique of cognitive nativism, specifically, the core knowledge hypothesis. My discussion begins with a brief summary of this hypothesis, followed by a discussion of Everett’s methodological concerns.

### The Core Knowledge Hypothesis

Core knowledge theory articulates a cognitive hypothesis regarding the origins of knowledge (for reviews, see Spelke, 1994; Carey and Spelke, 1996; Spelke, 2000; Spelke and Kinzler, 2007; Carey, 2009). It asserts that knowledge is constrained by domain-specific principles that are innate, and consequently, these principles tend to emerge universally, in early development. In addition, core knowledge plays a secondary role in guiding the acquisition of knowledge later in life. For example, young infants (e.g., Feigenson et al., 2002; Wynn et al., 2002; Izard et al., 2009; de Hevia et al., 2014) and animals (e.g., Uller et al., 2001; Flombaum et al., 2005) exhibit a rudimentary understanding of number based on principles that are apparently universal and innate. Unlike the early core knowledge of number, mathematical theory is neither innate nor universal, but its organization appears to follow on the heels of core knowledge principles. Other putative systems of core knowledge concern the representation of objects (e.g., Carey and Xu, 2001), space (e.g., Hermer and Spelke, 1994), others’ minds (e.g., Leslie, 2005) and morality (e.g., Hamlin et al., 2007, 2010). In all cases, infants tend to converge on narrow organizational principles that are apparently unattested in their experience, and these foundations guide the acquisition of related bodies of knowledge later in life (e.g., mathematics, physics, psychology and ethics).

Berent (2013a,b) suggests that phonology might present another core knowledge system, whose organization is constrained by universal grammar. This hypothesis predicts that speakers of all languages converge on shared phonological principles for which they lack inductive basis. To the extent phonological knowledge is abstract, it is possible for it to emerge across language modalities—in signed and spoken phonological systems. Finally, the view of phonology as a system of core knowledge further explains the close links between phonology and reading—a cultural invention that is obviously not universal, but is often phonologically based.

Everett rejects cognitive nativism outright. In his words “There is no human nature if by this we mean a kind of *a priori* knowledge common to all and only humans” (Everett, 2016a, p. 51)^1^. “There is nothing like instincts or modules in our higher-level cognitive abilities, e.g., language, interpretative principles of the world around us” (Everett, 2016a, p. 54). But Everett never discusses the details of any of the many experimental studies that demonstrate core knowledge in infancy. Instead, his objections (e.g., to a “moral instinct”) “boil down to three: (i) designer bias (i.e., the appeal to the notion that “humans are the way they are for reasons beyond their control,” p. 660); (ii) Ivy league bias (i.e., “assume the person at the most prestigious university is correct,” p.661); and (iii) simple answers to complex questions” (p. 660). None of these arguments speaks to the experimental evidence itself.

At a broader level, Everett (2016b) outlines two classes of in-principle objections to the core knowledge hypothesis. First, he is worried that this hypothesis is “difficult to falsify”. He next proceeds to attack cognitive nativism based on methodological, genetic and evolutionary considerations. I believe these concerns are unfounded, and I consider them next in turn. My goal here is to demonstrate that cognitive nativism, generally, and phonological nativism, specifically, remains a viable hypothesis. The specific evidence in its support is discussed in Section “A Case Study: The Restrictions on Syllable Structure.”

### Falsifying the Core Knowledge Hypothesis: The Cognitive Tests

Contrary to Everett’s worries, the core knowledge hypothesis makes some clear, readily falsifiable claims. This hypothesis would be readily falsified by showing that putative principles of core knowledge either (a) have no effect on behavior; (b) emanate from extraneous sources, external to the domain in question; or (c) are induced (i.e., learned) by tracking the statistical or structural regularities in the child’s experience. In the specific case of phonology, the hypothesis that a principle P forms part of a core phonological system (i.e., universal grammar) would be falsified by either showing that the preference attributed to P is inactive (1) (a), or by showing that it is neither linguistic or innate (i.e., it can be captured by sensorimotor constraints or ones that are induced from experience, see 1b–c).

(1) *Falsifying the core knowledge hypothesis*. The hypothesis that a principle P forms part of core knowledge of phonology (UG) will be falsified by showing that
  (a) P is inactive.
  (b) P emanates from extraneous non-phonological sources (e.g., sensorimotor constraints).
  (c) P is induced (i.e., learned) by tracking the statistical or structural regularities in the child’s experience.

To illustrate how one can apply these conditions, let us consider in detail one specific example, concerning the putatively universal constraint on syllable structure (i.e., ONSET, Prince and Smolensky, 1993/2004). To reiterate, my goal here is to demonstrate how this constraint can be falsified, *in principle*; I am not concerned with whether the ONSET hypothesis is correct.

ONSET is a putatively universal phonological principle (P) that requires syllables to have an onset (i.e., begin with a consonant). Accordingly, syllables like *pa* should be better formed than *ap*, resulting in a preference for *pa* across languages, in language processing and acquisition.

(2) *ONSET: Syllables must have onsets*.

The simplest way to falsify ONSET is to demonstrate that it is inactive in a given language (e.g., English). Notice that counter-examples (i.e., syllables like *ap, e.g., ap.pen.dec.to.my*) would not necessarily establish that fact. In modern phonological theory (e.g., Prince and Smolensky, 1993/2004; McCarthy and Prince, 1995), all grammatical constraints are violable (an assumption that is independently motivated; for discussion see Prince and Smolensky, 1993/2004; McCarthy and Prince, 1995), so the existence of syllables like *ap* does not necessarily show that ONSET is inactive. However, one could falsify this hypothesis by documenting a language that actively promotes onsetless syllables (i.e., a preference for *a* or *ap* over *pa*). Some authors have argued that Arrernte presents precisely this case (Breen and Pensalfini, 2001), although the matter remains a topic of controversy (Berry, 1998; Topintzi and Nevins, 2014).

**FIGURE 1:**
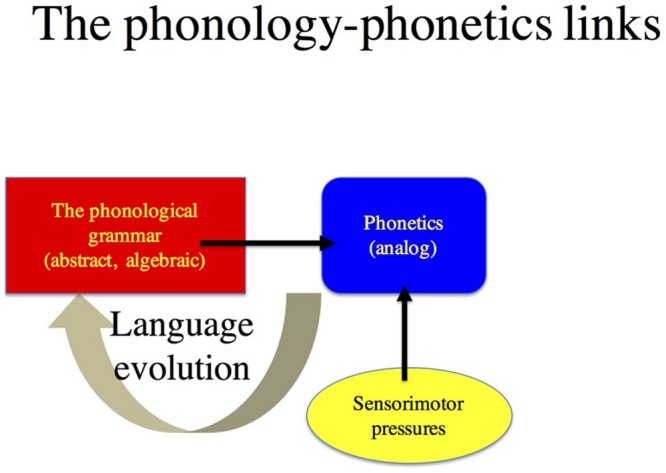
***The hypothesized links between phonology and the phonetic system*.** At the heart of the phonological system is the phonological grammar—a set of principles that are abstract and algebraic (as defined in Section Other Objections to *the Phonological Mind* Thesis), distinct from the phonetic system. Nonetheless, phonetic and sensorimotor pressures shape phonology in language evolution and its acquisition to favor phonological principles that optimize phonetic and sensorimotor pressures.

Another way to falsify ONSET is to show that the *pa>ap* preference is ***causally*** determined ***solely*** by the relative ease of the perception and articulation of *pa.* The distinction between a sole direct cause and indirect pressures is absolutely crucial. Many phonological systems conspire to favor forms that optimize speech perception and production—this is precisely what would be expected if phonology were an adaptive biological system (Berent, 2013a,b). But correlation is not causation. *The Phonological Mind* hypothesis states that sensorimotor pressures constrain the phonological system not in on-line language processing, but rather off-line, in phylogeny—by favoring the biological evolution of abstract universal rules that optimize sensorimotor pressures (e.g., by natural selection, see **Figure 1**). ONSET presents one such putative constraint. By hypothesis, the ONSET constraint is abstract, so its effect should be independent of the articulatory and acoustic demands associated with the production and perception of specific sounds in a given context (e.g., in clear speech vs. noise), their modality of presentation (e.g., they should be active for both spoken and printed words), and the state of sensorimotor systems (typical or impaired). Demonstrating that phonological principle P can be subsumed by sensorimotor constraints would falsify the hypothesis that P forms part of the core knowledge system of phonology.

Learnability presents a third critical test of the hypothesis. A principle P would be falsified if the relevant generalization were induced from experience with similar forms. For example, ONSET would be falsified if the preference for *pa* (over *ap*) *required* experience with syllables like *pa, ba, ma*, etc. As in the case of sensorimotor correlates, the role of experience must be interpreted with caution. And indeed, the (un)learnability of P does not mean that its acquisition requires no experience at all. It is trivial to show that, absent some minimal triggering conditions, language will not emerge normally. Similarly, absent experience with speech, deaf individuals will develop a sign language, whose phonology is distinct from that of hearing speakers. Experience, then, clearly plays a critical role in ***triggering*** the unfolding of the putative phonological phenotype (Berent, 2013a, p. 245). But triggering should not be confused with the cognitive process of inductive learning. The triggering condition entails that the *pa* preference might require experience with speech, generally. By contrast, induction means that the *pa* preference will require experience with ***specific*** types of syllables—ones whose structural properties overlap with those of the preferred *pa* type (e.g., labial-initial syllables, such as *pa, ba*). The hypothesis that certain phonological primitives and constraints are innate cognitive traits (i.e., they arise in the normal course of development, but do not result from a cognitive process; Samuels, 2004) explains this fact.

### Evaluation of Innateness at the Behavioral, Genetic and Evolutionary Levels

Our discussion so far shows that the hypothesis of core knowledge is clearly falsifiable, and I believe Everett and I agree on the criteria outlined in (1). In his view, however, the list of methodological requirements for demonstrating core knowledge is more extensive (see 3). In particular, Everett imposes restrictions on the range of admissible experimental methods, and on the genetic and evolutionary conditions for demonstrating core knowledge. I consider these three objections in turn.

(3) *Everett’s conditions for demonstrating that principle P forms part of a core knowledge* (based on Everett, 2016b, p. 4):
  (a) Demonstrate the role of P in infancy by relying on methods “more sound than babies’ sucking or eye-movements.”
  (b) “Keep genetics and epigenetics (constraints – embryological, environmental – on the strength, absence, or presence of genetic effects) separate.”
  (c) Provide a plausible account for the evolution of the trait.

Everett’s first requirement stipulates conditions on the behavioral methods admissible for demonstrating core knowledge. In his opinion, the support for core knowledge cannot be based on measures involving sucking or eye movement. This requirement arbitrarily rejects a very large literature that demonstrates principles of core knowledge in the first months of life. Everett (2016b) provides no justification for this requirement. Elsewhere (Everett, 2016a, p. 664), he notes the concern that the infant “is acting upon information that is different from the information the researcher is thinking about.” While Everett is of course, right that the link from behavior to cognitive structure is indirect, this challenge is certainly not specific to looking time or sucking methods. *Any* response to contrasting stimuli—be it manual button press, looking time or neural activity—can only be linked to cognitive structure if those stimuli are matched for all other relevant dimensions. The critical challenge for making such inferences is the control of stimuli, not the modality of response. However, Everett does not show that such controls are lacking in any of the hundreds of studies that employ this procedure. In fact, none of these experimental results is reviewed with any detail. In any case, the evidence for core knowledge in infancy is not limited to looking time or sucking methods. For example, near infrared spectroscopy shows that the brain activity of neonates is modulated by the syllable hierarchy (Gómez et al., 2014). Furthermore, the results from looking time methods are corroborated by findings from brain measures (cf., the evidence for rule learning in behavioral vs. brain measures, in Marcus et al., 1999 and Gervain et al., 2012). Accordingly, the exclusion of the great majority of the infant literature on core knowledge has no justification.

Everett’s next condition requires that one furnish genetic evidence for core knowledge, and in so doing, one must “keep genetics and epigenetics (constraints – embryological, environmental – on the strength, absence, or presence of genetic effects) separate.” To illustrate the logical difficulties inherent in the notion of innate knowledge, Everett considers the “pro-drop” parameter (i.e., optional deletion of the subject, as in “He left”). By his account, each of the two settings of the parameter (i.e., whether or not the subject can be dropped) must each be associated with different genes. Accordingly, if the “pro-drop” parameter were truly innate, then this knowledge must be “located somehow, somewhere in the human genome” (p. 2), and consequently, one would have expected these two settings to doubly dissociate: some genetic mutations should have selectively impaired the acquisition of a “pro-drop” language, but spared a language that lacks the “pro-drop” option; other mutations should have produced the complementary pattern. However, no such dissociations are reported. Beyond this empirical challenge, Everett is concerned that the hypothesis of innate knowledge presents an inherent contradiction. If knowledge (e.g., the “pro-drop” parameter) is genetically coded, then it must be subject to mutations, which would ultimately prevent certain languages from being learnable. In the case of the “pro-drop” parameter, Everett would predict that some language learners should be genetically predisposed toward favoring the “drop” option, whereas others (those carrying the opposite parameter setting) should be predisposed toward learning languages that do not drop their subject. The result is that “not all people may be able to learn every language” (p. 2). Put differently, if UG is innate, then it cannot be universal.

However, there are a number of fallacies in this logic. First, innateness and universality are not an oxymoron. Humans are innately equipped with two eyes, yet this trait does not become abruptly extinct (i.e., non-universal) in the population by sudden spontaneous mutations. This is because innate traits are buffered by homeostatic mechanisms that seek to detect and correct replication errors in the genome, as well as cell and tissue malfunctions during embryonic development. These checks and balances are not infallible. Accordingly, rare genetic abnormalities or developmental problems (like the loss of particular cell types or interference from external factors that embryos can be exposed to, such as alcohol, nicotine, synthetic steroid hormones, etc.) could prevent the typical acquisition of universal grammar, just as they can result in a single “cyclopean” eye in human fetuses (Sarnat et al., 2014). Such rare cases of dysfunction do not throw into question the reality of the complex sequence of developmental events that forms the innate basis of the “two eyes” trait and its universality in the normal population. The same holds for universal grammar.

Similarly, there is no biological reason that distinct settings of a UG principle (e.g., “pro-drop”) must doubly dissociate. Everett predicts such dissociations because he believes that distinct innate principles (e.g., the two settings of the “pro-drop” parameter) must be “localized” in distinct bits of DNA. But this requirement is based on a misunderstanding of the relation between *genotypes* and *phenotypes*. Core knowledge (e.g., UG) concerns properties of the cognitive *phenotype*—the putative set of cognitive principles that are somehow represented in the brains of individual speakers; this is not conceptually identical to the *genotype* of these individuals (e.g., their particular sequences of DNA bases), nor are genotypes and phenotypes necessarily related in a one-to-one fashion. By hypothesis, the tendency of humans to converge on a cognitive phenotypic trait is constrained by their genome, but the links between the phenotype and genotype are complex. For example, the “blue eye” and “brown eye” phenotypes are not expressed on distinct genes; rather, eye color is a complex genetic trait that is linked to multiple single nucleotide polymorphisms that do not sum up in a linear, additive fashion (e.g., Liu et al., 2009). Likewise, the expression of innate traits is inextricably linked to epigenetic factors (Ridley, 2003) as well as chance events (Balaban, 2006). Language (e.g., the “pro-drop” parameter) is unlikely to form an exception.

None of the above, however, means that the hypothesis of (innate) core knowledge is vacuous or untestable. Rather, these facts remind us that innateness is a multifaceted problem that can be evaluated at different levels of analysis. Genetics and the cognitive sciences represent two very different levels. Critically, it is both unreasonable and biologically unrealistic to expect direct, one-to-one mapping across these levels (Balaban, 2006). The problem of collapsing across levels of analysis is hardly unique to the cognitive sciences. Chemical reactions, for instance, attain different explanations in the fields of physics and chemistry, and it is impossible to reduce one level of analysis to the other (Chomsky, 2014). In short, scientific hypotheses can be evaluated *only at the level of analysis to which they apply* (Fodor and Pylyshyn, 1988; Samuels, 2004; Chomsky, 2014; see also Berent, 2013a; Embick and Poeppel, 2015), and in the case of core knowledge, this is squarely within the cognitive level. Everett would be entirely right to expect cognitive scientists to separate the role of innate principles and learning at the *cognitive* level. However, it does not follow that each cognitive trait will be transparently mapped to distinct bits of DNA, irrespective of epigenetic factors. Everett’s insistence on “locating” UG principles in the genome and “separating genetics from epigenetics” is a red herring, rooted in the erroneous conflation of the cognitive and genetic levels of analysis.

Everett’s final condition, his demand for a complete evolutionary history of core knowledge, is another such diversionary tactic. Everett is of course right to urge us to explore the evolution of core (phonological) knowledge—a question Berent (2013a) examines by conducting an in-depth analysis of the precursors of phonological principles in non-humans (see Chapter 10). But a detailed evolutionary history is by no means a logical ***prerequisite*** for establishing that core knowledge exists. To reiterate, the core knowledge hypothesis is first a claim concerning the synchronic cognitive state of individual speakers. We now turn to evaluating the empirical evidence for its support in the case of phonology.

## A Case Study: The Restrictions on Syllable Structure

*The Phonological Mind* examines the origins of phonological primitives and constraints by reviewing the empirical evidence in multiple case studies using a broad interdisciplinary perspective, informed by linguistic analysis, behavioral experiments with adults and children, and neuroimaging studies. In most of these cases, the conclusions are unclear—a fact that I openly acknowledge (contrary to Everett’s assertions). One such case, however, has been the subject of systematic investigation that directly pits the UG account against various non-linguistic explanations (per the requirements in 1a-c above). Accordingly, this case features prominently in Berent (2013a) and it forms the center of Everett’s (2016b) critique. I will first describe the linguistic phenomenon at hand and the experimental evidence, and then consider Everett’s objections.

### The Linguistic Phenomenon

Across languages, certain syllable types are systematically preferred to others. Syllables like *bla*, with stop-liquid onsets, are preferred (e.g., more frequent) relative to *bna* (i.e., stop-nasal combinations), which, in turn, are preferred to *bda* (two stops); least preferred are syllables like *lba* (i.e., liquid-stop sequences); together those preferences give rise to a syllable hierarchy (see 4). Such preferences have been documented statistically in the distribution of these syllables in language surveys (Greenberg, 1978; Berent et al., 2007), so their existence is well established. The question is whether those facts reflect linguistic principles that are active universally, in the grammar of every individual speaker.

(4) The syllable hierarchy: blif>bnif>bdif>lbif

Everett strategically diverts the discussion of this cognitive question by plunging into a technical linguistic controversy concerning one formal analysis of this phenomenon, the sonority sequencing generalization (SSG, for definition, see **Box 1**; see also Saussure, 1915/1959; Vennemann, 1972; Hooper, 1976; Kiparsky, 1979; Steriade, 1982; Selkirk, 1984; Clements, 1990; Gouskova, 2001; de Lacy, 2004; Smolensky, 2006; Zec, 2007). In so doing, he hopes to discredit the hypothesis that the syllable hierarchy is universal. Everett asserts that sonority is a circular concept that is devoid of phonetic basis, and he marshals a number of challenges to this analysis. But many of these challenges are erroneous (see **Box 1**). His exposition of the linguistic literature (based on Ohala’s seminal work from the Ohala, 1990) ignores more recent developments in phonetics (Parker, 2002) and phonology (e.g., Parker, 2012, and chapters therein). Furthermore, Everett overlooks the fact that the SSG correctly generates additional predictions regarding several other syllable hierarchies (see 6), and these predictions are borne out by both linguistic and experimental evidence (e.g., Berent et al., 2009, 2010, 2011b, 2012a; Lennertz and Berent, 2015; Tamasi and Berent, 2015).

**Box 1. Everett’s critique of sonority.**

An influential account of the syllable hierarchy appeals to *sonority*. Sonority is an abstract phonological property that correlates with the loudness of phonological elements (Clements, 1990; Zec, 2007; Parker, 2008, 2012). On a basic sonority scale (Clements, 1990), the most sonorous (e.g., loudest) consonants are glides (e.g., *w, y*; with a sonority (s) of 4), followed by liquids (e.g., *l, r*; *s* = 3), nasals (e.g., *m, n*; *s* = 2) and obstruents (e.g., *b, p*; *s* = 1). Using these levels, one can calculate the sonority distance in the onset by subtracting the sonority level of the first onset consonant from that of the second (Δ*s* = s2-s1, see 5). Accordingly, syllables like *bla* exhibit a large rise in sonority (*s* = Δ2) and *bna* exhibits a smaller rise (Δ*s* = 1), whereas *lba* exhibits sonority fall (Δ2 = -2). Indeed, as the sonority cline decreases, the syllable becomes underrepresented across languages (Berent et al., 2007). Thus, *blif*, for instance, is more frequent than *bnif*, which is more frequent than *bdif;* least frequent are syllables like *lbif.*

(5) The sonority distance in complex onsets
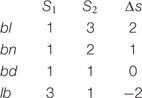

This observation is captured by a constraint on syllable structure known as the ***sonority sequencing generalization*** (SSG). The SSG states that onsets must rise in sonority, whereas codas must exhibit a sonority fall. A related principle, the ***minimal sonority distance***, states that languages restrict the minimal sonority distance they allow; English, for instance, requires a rise of at least two steps, so it allows syllables like *bla (*Δ*s = 2*), but bans syllables like *bda* (Δ*s* = 0). Smolensky (2006) further shows that the preference for syllables with large sonority distances can be traced to putatively universal constraints.

Everett amasses several challenges to the SSG; some are justified, but most are not. For example, Everett notes that the sonority hierarchy does not explain the ban on English syllables such as *bwa*, or account for the aversion to syllables like /ji/ across languages. However, these observations are fully in line with the SSG. Considering the English ban on *bwa*, Everett is right to note that the SSG should render such a syllable well formed in English (as its sonority distance, Δ*s* = 2, is comparable to *twin*, for instance). However, this does not show that the SSG is wrong. Indeed, no single phonological constraint can single-handedly capture the entire phonology. In the case of *bwa*, its avoidance is due to an independently motivated constraint that bans adjacent identical features (i.e., the adjacent labials in *bwa*, which are banned by the Obligatory Contour Principle, McCarthy, 1994), so this fact has no bearing on the SSG. The second observation—the aversion to /ji/ across language—actually *follows* from the SSG. Because these glide-vowel sequences exhibit a minimal sonority rise, they are expected to be dispreferred. These facts present an embarrassment to Everett’s linguistic analysis, not to the SSG.

Other facts (e.g., the allowance of English syllables like *spot*), however, are known counterexamples to the SSG. Since English typically requires its syllables to exhibit a rise of two steps in sonority, plateaus such as *spot* should have been unattested. While this fact is amenable to a formal explanation (Selkirk, 1982), *s*-initial onsets present a systematic counter-example to the sonority hierarchy in many languages, and, contrary to Everett’s accusations, I have openly acknowledged these challenges in print (Berent et al., 2007; Berent, 2013a, p. 167).

(6) Other syllable hierarchies predicted by the SSG:
  (a) mla>mda
  (b) fsa>fta

These challenges notwithstanding, the concept of sonority is still attractive because it generates a number of correct predictions regarding several other hierarchies. For example, the sonority hierarchy correctly predicts a preference for nasal-sonorant over nasal-stop onsets (e.g., for *mlif* over *mdif*). Similarly, a more detailed (and independently motivated, Steriade, 1982) sonority scale that renders fricatives more sonorous than stops predicts a preference for *fsik* (a fricative-fricative onset, i.e., a sonority plateau) over *ftik* (a fricative-stop onset, i.e., a very small fall in sonority). These results are borne out by experimental evidence (e.g., Berent et al., 2009, 2010, 2011b, 2012a; Lennertz and Berent, 2015; Tamasi and Berent, 2015). For reasons of space, my discussion of the sonority hierarchy focuses only on the stop-sonorant sequence in (1), but these other hierarchies should be kept in mind.

While there is much evidence to suggest that the SSG merits careful consideration, it is important to keep in mind that the SSG and the syllable hierarchy are **not** one and the same. The syllable hierarchy is a hypothesis concerning speakers’ grammatical ***preferences***; the SSG presents a *particular* account of the ***formal mechanisms*** that give rise to these preferences. Other formal accounts, however, can capture the hierarchy without appealing to sonority (e.g., Smolensky, 2006, and its discussion in Berent, 2013a, pp. 172–174). So even if the SSG should turn out to be wrong (a question that is still very much open), the syllable hierarchy could still remain viable. It is the syllable hierarchy, then, not the SSG, that is the topic of our discussion. We now turn to examining the relevant experimental evidence.

### The Experimental Evidence

My colleagues and I have examined the hypothesis that the syllable hierarchy (as in 4), as well as the related hierarchies (see 6) is the product of universal grammatical constraints (Berent et al., 2007, 2008, 2009, 2010, 2011a,b, 2012a, 2013b, 2014, 2015; Gómez et al., 2014; Lennertz and Berent, 2015; Tamasi and Berent, 2015; Zhao and Berent, 2015).

Our research program proceeds in two steps. First, we ask whether speakers are sensitive to the structure of syllables that they have never heard before. For example, do English speakers favor *bnif* to *bdif*, and *bdif* to *lbif* despite the fact that none of these syllable types exists in their language? To the extent that such preferences are detected, we next examine whether they result from universal grammatical constraints, or from various non-grammatical sources—auditory, articulatory, or lexical.

(7) Testing the syllable hierarchy:
  (a) Ill-formed syllables are systematically misidentified—the worse formed the syllable, the more likely its misidentification.
  (b) The misidentification of ill-formed syllables is inexplicable by non-grammatical sources, including:
    (i) Auditory/phonetic failure
    (ii) Articulatory difficulty
    (iii) Similarity to familiar syllables

**FIGURE 2:**
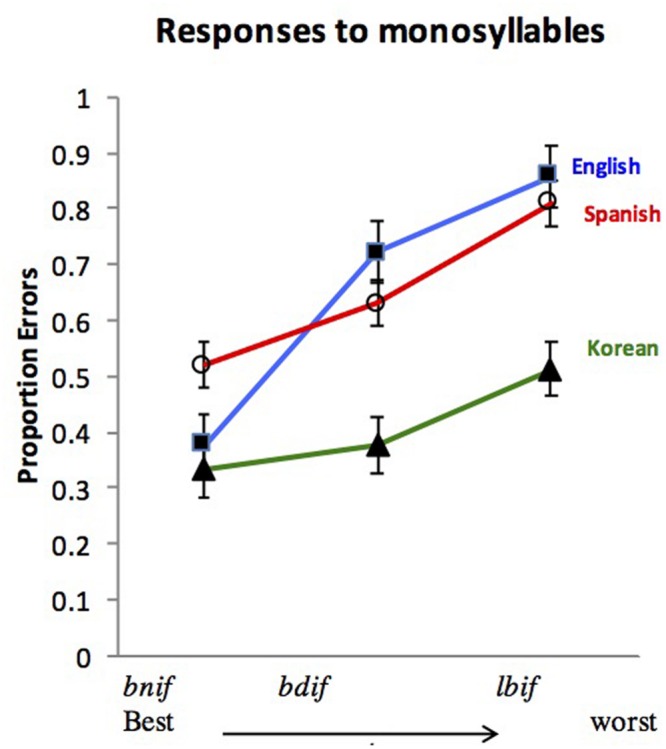
**The effect of the syllable hierarchy on responses to monosyllables by speakers of English, Spanish, and Korean.** Participants in this task indicate whether the auditory input has one syllable or two. Results show that worse-formed syllables elicit more errors. Error bars are confidence intervals for the difference between the means. Data from Experiment 1 in Berent et al. (2007, 2008, 2012b).

To infer people’s preferences, we exploit the phenomenon of *grammatical repair*. We reason that, if all grammars universally encode the syllable hierarchy, then ill-formed syllables will not be represented faithfully. Instead, ill-formed syllables (e.g., *lbif*) will be recoded (i.e., repaired) by the grammar as better-formed syllables (e.g., as *lebif*). Repair, then, can potentially provide a litmus test for the ill-formedness of a syllable. And if ill-formedness depends on the syllable hierarchy, then this hierarchy should determine the likelihood of repair—the worse formed the syllable, the more likely its repair, hence, its misidentification. These predictions are borne out by numerous studies with speakers of various languages. **Figure 2** summarizes results from speakers of English (Berent et al., 2007), Spanish (Berent et al., 2012b) and Korean (Berent et al., 2008).

**FIGURE 3:**
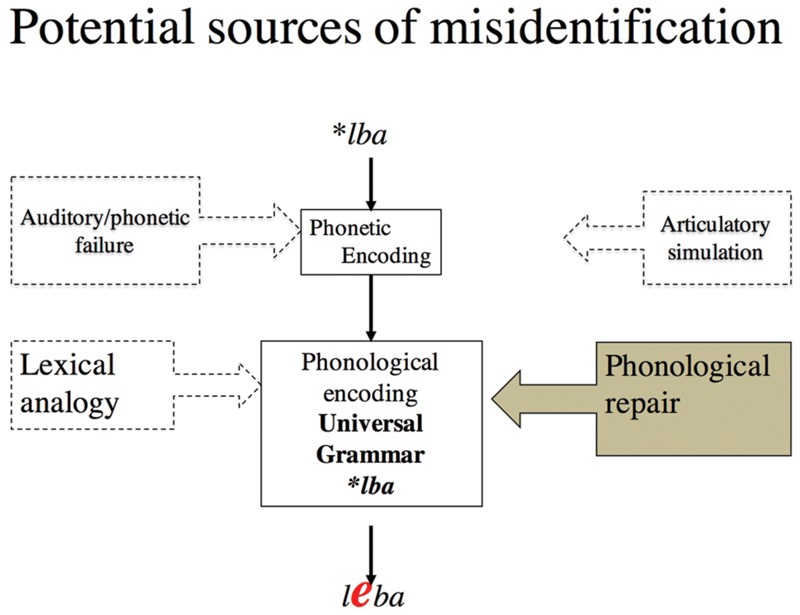
**Potential sources of misidentification.** According to the phonological account, ill-formed syllables (e.g., lba) are misidentified because they are repaired by the grammar due to their violation of universal grammatical constraints. Misidentification, in this view, is due to phonological repair, occurring during phonological encoding. However, by alternative accounts, misidentification results from the dissimilarity of the input to the lexicon, or difficulties in its phonetic encoding, due to failure either to extract its auditory/phonetic form, or to covertly simulate its articulation.

Obviously, misidentification can also emerge from many other sources (see **Figure 3**). In particular, syllables like *lbif* could be misidentified because people fail to extract their phonetic form from the auditory signal (Davidson and Shaw, 2012; Wilson et al., 2014). Similarly, syllables like *lbif* might exert greater articulatory demands. Given the known links between speech perception and articulatory action (Fadiga et al., 2002; Pulvermüller et al., 2006), the misidentification of *lbif* in perception could result from difficulties in its covert production (Redford, 2008). Finally, it is conceivable that the syllable hierarchy in (4) is encoded in the phonological grammar of *some* speakers due to experience with similar syllables in their language (e.g., Daland et al., 2011). Accordingly, the attribution of misidentification to core knowledge of phonology (i.e., UG) can only be done by elimination—after rejecting non-phonological (acoustic and articulatory) and inductive sources, as indicated in (1) above.

**FIGURE 4:**
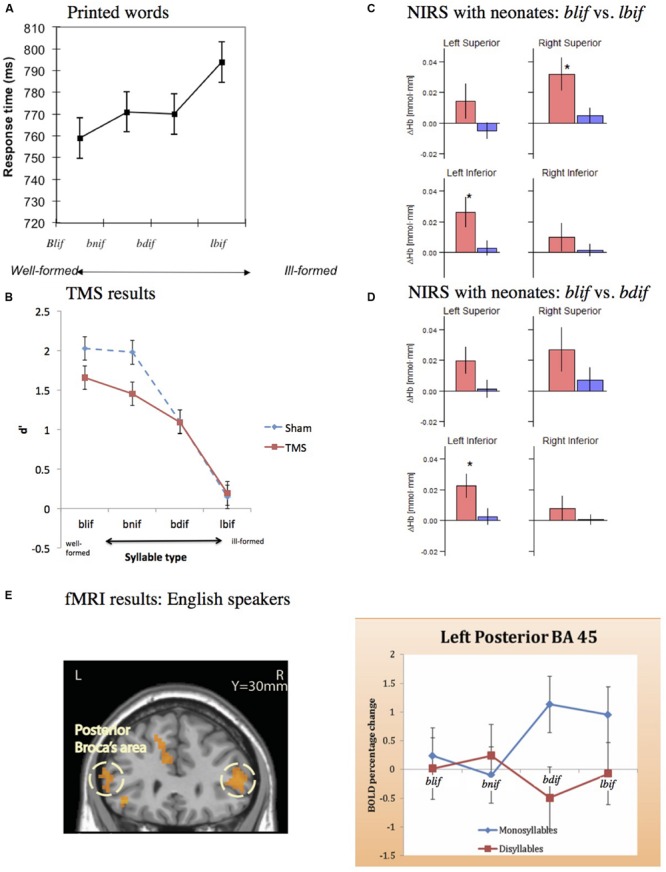
**Tests of non-grammatical explanations for the misidentification of ill-formed syllables. (A)** Sensitivity to the syllable hierarchy with printed materials. In this task, people make identity judgment (e.g., is *lbif* = LEBIF?). Results show that, as the syllable becomes worse formed, people take longer to discriminate monosyllables from their disyllabic counterparts. **(B)** Sensitivity to the syllable hierarchy despite suppression of the lip motor by TMS. Results show that TMS attenuates overall discrimination (d′), but it does not diminish the sensitivity to the syllable hierarchy. **(C,D)** Sensitivity to the syllable hierarchy in neonates. Results plot the effect of the syllable hierarchy on differences in oxyhemoglobin (red) and deoxyhemoglobin (blue) concentration changes for ill-formed minus well-formed syllables, contrasting either **(C)** sonority rises and falls (e.g., *blif* vs. *lbif*) or **(D)** sonority rises and plateaus (e.g., *blif* vs. *bdif*) at four regions of interest (superior and inferior at the left and right hemisphere; ^*^ indicates significant differences, p < 0.05). **(E)** The effect of the syllable hierarchy on the posterior part of Broca’s area: as the syllable becomes ill-formed, activation increases (data from Berent et al., 2014).

My colleagues and I have addressed each of these alternative explanations in turn. Contrary to the phonetic/auditory explanation, we found that ill-formed syllables do not present greater processing cost under conditions that promote attention to the phonetic properties of the stimuli (e.g., Berent et al., 2007, Experiments 5–6; Berent et al., 2012a). This finding challenges the assertion that such syllables are misidentified because they are harder to encode at the phonetic/auditory level. Furthermore, the (phonological) difficulties in processing ill-formed syllables persist when the stimuli are presented in print—in the absence of any phonetic/auditory demands whatsoever (see **Figure 4A**; Berent, 2008; Berent et al., 2009; Berent and Lennertz, 2010; Tamasi and Berent, 2015).

Our results are likewise inconsistent with an articulatory explanation. If the difficulties in processing ill-formed syllables result from difficulty in their subvocal articulation, then procedures that suppress the articulatory motor system should attenuate the disadvantage of ill-formed syllables. Contrary to the articulatory account, however, we found that ill-formed syllables are misidentified even when articulation is suppressed—either mechanically, by having participants bite on tongue depressors (Zhao, 2015), or electromagnetically, by disrupting the lip motor area in the brain using transcranial magnetic stimulation (Berent et al., 2015). Additional evidence for an abstract grammatical locus of repair is presented by neurological patients, whose repair of onset clusters in production is demonstrably distinct from sensorimotor pressures (Buchwald et al., 2007; Buchwald, 2009; Cohen-Goldberg et al., 2013; Miozzo and Buchwald, 2013).

Finally, several studies counter the lexical analogy explanation—the possibility that *bnif* is preferred solely for its similarity to *sniff*, for instance. We find that sensitivity to the syllable hierarchy obtains even in languages that arguably lack complex onsets of any type (for Korean see Berent et al., 2008; for Mandarin Chinese, Zhao and Berent, 2015), and even in the absence of any lexicon at all—among neonates (Gómez et al., 2014, see **Figure 4**). These findings from neonates agree with the outcomes from an imaging study with adults (Berent et al., 2014) showing that ill-formed syllables elicit a monotonic increase in the activation of the posterior part of Broca’s area (BA 45, see **Figure 4D**). Together, these results suggest that syllable structure is constrained by abstract grammatical principles that are broadly shared across languages, possibly universally. The repair of ill-formed syllables results from their violation.

### Everett’s Objections to the Empirical Findings

Given the large experimental literature that has examined the syllable hierarchy, one would expect a critique to scrutinize the experimental logic, methods, statistical analysis or results of specific studies. However, Everett (2016a,b) cites none of those primary sources. In fact, he hardly acknowledges that any of these studies has even *attempted* to address any non-grammatical explanation for the results. Instead, his exposition reduces the experimental logic *ad ridiculum* to the following: if people misidentify *lbif*, ergo, the SSG is an instinct.

Patient readers must endure the bulk of his critique to learn that the actual rationale guiding the experimental investigation is far more nuanced, and those non-phonological (and non-universal) sources for the syllable hierarchy have been carefully considered and evaluated experimentally. Even then, however, Everett’s description hardly does justice to the research program as a whole. For example, he briefly acknowledges that the syllable hierarchy has been replicated in silent reading (contrary to the auditory/phonetic account), but then immediately moves to dismiss these results on grounds that “we know too little about the relationship between speaking and reading” (Everett, 2016b, p. 7). These alleged gaps, however, do not stop Everett from offering an original reading theory of his own (Everett, 2016b, pp. 7–8; italics mine):

...in looking at new words speakers often try to create the phonology in their heads and so this “silent pronunciation” could guide such speakers’ choices, etc. Everyone (modulo pathology) has roughly the same ears matched to roughly the same vocal apparatus. Thus although phonologies can grammaticalize violations of functionally preferable phonotactic constraints, one would expect that in experiments that clearly dissociate the experimental data from the speaker’s own language, the functionality of the structures, e.g., being auditorily easier to distinguish, will emerge as decisive factors, accounting for speakers’ reactions to non-native sequences that respect or violate sonority sequencing, etc. In fact, there is a name for this, though with a somewhat different emphasis, in Optimality Theoretic Phonology (Prince and Smolensky, 1993/2004; McCarthy and Prince, 1994) – the “emergence of the unmarked.”

This passage is striking for various reasons. First, it is puzzling to see the liberty Everett takes in dismissing the huge literature on reading, which suggests (based on behavioral results and evidence from neuroimaging) that speakers are acutely sensitive to the phonological structure of printed words (for reviews: Perfetti, 1985; Van Orden et al., 1990; Berent and Perfetti, 1995). Reading, to be sure, is not the only experimental literature to be singlehandedly rejected by Everett—recall that he also dismisses the entire infancy literature of sucking/looking time. This is a peculiar move from a serious scholar who is committed to “empirical adequacy.”

Moving to Everett’s own reading theory, it is unclear how, in this proposal, auditory constraints come to shape speakers’ behavior. Surely, people cannot possibly misidentify the printed word *lbif* because this *stimulus* imposes excessive auditory demands; perhaps Everett is suggesting that people *estimate* the demands auditory stimuli *might* impose, and use this estimate to inform their judgments. However, there is no evidence that such a mechanism exists, let alone that it can estimate the demands of unfamiliar stimuli. Either way, misidentification, by this account, would reflect the outcome of a process that *estimates* the demands on speech perception, not a *perceptual* mechanism (auditory or phonetic). Alternatively, perhaps the syllable hierarchy reflects principles that are inferred on the fly. Everett implies that a single encounter with an unfamiliar stimulus results in the emergence of phonological constraints, a phenomenon he attributes to the *The Emergence of the Unmarked* (referring to a central outcome of Optimality theory, McCarthy and Prince, 1994). But *The Emergence of the Unmarked* (at least according to McCarthy and Prince, 1994) is due to innate, universal grammatical constraints, not auditory difficulties. And if such constraints were active, then this would support the UG account, rather than disprove it.

In any case, the existing experimental evidence against the auditory account is not limited to findings from printed materials. Other results show that, when prompted to attend to phonetic detail, people do not necessarily experience greater difficulties with ill-formed syllables (Berent et al., 2007, 2012a). Likewise, individuals with dyslexia, whose auditory and phonetic systems are demonstrably impaired, exhibit intact sensitivity to the syllable hierarchy (Berent et al., 2013b, 2016). These two sets of results are important because they suggest that the misidentification of ill-formed syllables is rooted in the phonological, rather than the phonetic or auditory systems.

### Summary

Everett’s critique centers on a specific formal analysis of the syllable hierarchy (SSG), not the evidence for the hierarchy itself, and for the most part, his formal objections are erroneous. His only objection to the experimental findings is based on an incoherent model of silent reading for which there is no support. I thus conclude that Everett’s attack on the syllable hierarchy is unfounded.

## Other Objections To *The Phonological Mind* Thesis

Anticipating the shortcomings of his own attack on the syllable hierarchy, Everett declares that even if the SSG were to be replaced by more adequate formal principles, the “core knowledge” hypothesis would still crumble. This is because “the evidence she (IB) provides for an instinct fails no matter what principle she might appeal to” (p. 7). In support of this assertion, Everett submits that all other hallmarks (actual or presumed) of core knowledge in phonology need not arise from any innate universal principles. Specifically, he asserts that algebraic rules can be learned from experience, and that the properties of phonological systems (unique shared design, early onset, and spontaneous regenesis) can all be traced to sensorimotor, rather than phonological pressures. The regenesis of signed phonological systems, in Everett’s view, is irrelevant to spoken phonology, and the early onset of spoken phonology simply reflects rapid learning. Finally, scaffolding (the propensity of phonological systems to give rise to reading) is simultaneously false and non-specific to phonology. Once again, however, Everett’s anti-nativist fervor clouds some critical scientific nuances.

(8) Other evidence against core knowledge of phonology:
  (a) Algebraic rules are learned from experience.
  (b) The unique, shared design of phonological systems, their early onset in development and their spontaneous regenesis are all explained by sensorimotor pressures, not principles specific to phonology.
  (c) Regenesis establishes a false analogy between signed and spoken phonology.
  (d) The early onset of phonology reflects early learning.
  (e) Scaffolding is both false and not specific to phonology.

Everett’s attack on algebraic rules conflates two very different hypotheses about (a) the computational properties of linguistic rules (i.e., the algebraic hypothesis) and (b) the origins of some rules (i.e., the Universal Grammar hypothesis), which together form the thesis of *The Phonological Mind* (see 9).

(9) The Phonological Mind thesis:
  (a) *The algebraic hypothesis* (Computation): The phonological grammar consists of algebraic rules.
  (b) *The universal grammar hypothesis* (Origins): Some grammatical primitives and constraints are innate.

The algebraic hypothesis asserts that phonological principles apply over abstract categories (e.g., “consonant”) rather than their members (e.g., the consonant *b* or *k*), such that all category members are treated alike (i.e., they form equivalence classes). Furthermore, the grammar encompasses mechanisms that operate over entire categories using variables (e.g., X, standing for “any consonant”) and, consequently, grammatical rules can encode abstract relations among categories (e.g., identity, recursion). For example, the ^∗^XXY rule (where X and Y stand for any two consonants) bans any XXY form, that is, any tri-consonantal structure with identical consonants at its beginning, regardless of whether these consonants are familiar or novel. The hypothesis that some grammatical rules are innate (i.e., the universal grammar hypothesis) is a second hypothesis that is logically distinct from the algebraic hypothesis. Everett attacks the algebraic hypothesis because algebraic rules, such as the Semitic ban on XXY stems (Greenberg, 1950), are learned in many cognitive domains. However, the algebraic hypothesis makes no claims about the innateness of any particular phonological rule or its domain specificity. Everett’s attack confounds the algebraic and UG claims.

The algebraic hypothesis is critical for two reasons. First, it explains productivity—the capacity of the grammar to generalize phonological principles across the board, to any member of a class (e.g., even to non-native consonants). Second, it allows one to draw a principled distinction between the (algebraic) phonological grammar, and the (non-algebraic, analog) phonetic and sensorimotor systems (see **Figure 1**). This distinction becomes particularly significant in light of Everett’s subsequent argument that phonological pressures *are* sensorimotor. Obviously, if phonological systems were to directly reflect analog sensorimotor pressures, then, *ipso facto*, such systems could not possibly be phonological (i.e., algebraic); they would be sensorimotor. Nonetheless, Everett is right to note that many phonological principles “conspire” to favor structures that optimize sensorimotor pressures. *The Phonological Mind* captures both the distinction between these two systems (phonology and the sensorimotor system) as well as their convergence. The distinction between the phonology and the sensorimotor systems is explained by the hypothesis that the two systems are distinct, and they differ in their computational properties. Their convergence is explained by the hypothesis that sensorimotor pressures shape the design of the phonological system in phylogeny (Berent, 2013a,b, see **Figure 1**). To reiterate, the correlation between phonology and sensorimotor pressures does not necessarily mean causation affecting on-line, language processing.

The hypothesis that some phonological principles are both algebraic (i.e., abstract and amodal) and innate would explain the capacity of phonological systems to emerge spontaneously in a new linguistic modality, in nascent sign language (i.e., regenesis: see 8c, e.g., Sandler et al., 2011; Brentari et al., 2012; Coppola and Brentari, 2014). The same hypothesis would also account for the early emergence of phonology in typical language development.

Everett rejects the evidence from regenesis because “no one has ever successfully demonstrated that signed languages have “phonology” in the same sense as spoken languages” (p. 8). It is unclear what Everett means by “successfully,” as there is certainly ample research to suggest that significant cross-modal phonological similarities exist. For example, as with spoken languages, signers encode syllables and constrain their internal structure by appealing to their sonority (defined by the visual salience of phonological features, e.g., Perlmutter, 1992; Brentari, 1993; Sandler, 1993; Williams and Newman, 2016). Furthermore, experimental evidence shows that English speakers spontaneously extend their phonological knowledge of syllable structure to signs (Berent et al., 2013a). Clearly, phonology is not confined to spoken languages.

Everett’s next stipulation, that the early onset of phonology in typical development is entirely due to rapid learning (8d), is likewise countered by evidence for the syllable hierarchy in neonates (Gómez et al., 2014). While knowledge seen at birth can be learned—fetuses are known to extract rhythmical properties of their maternal language *in utero* (e.g., Kisilevsky et al., 2009)—it is unlikely that such learning would extend to specific consonantal features. Indeed, infants do not show knowledge of the specific phonetic contrasts of their mothers’ languages until the end of their first year of life (e.g., Eimas et al., 1971; Werker and Tees, 1984). The findings of Gómez et al. (2014) do not establish what principles guide neonates’ preferences (e.g., phonological vs. phonetic). Nonetheless, neonates’ sensitivity to syllable structure is likely innate.

Moving to his last point (8e), Everett rejects scaffolding—the hypothesis innate core knowledge forms the basis for related bodies of knowledge that are acquired in later development. For example, the (innate, universal) core knowledge of number forms the basis for the recursive number system that emerges optionally in later development. In a similar vein, the putative innate knowledge of phonology could explain the fact that mature writing systems tend to encode phonological principles, and reading tends to recover phonological structure from print.

Everett objects on grounds that scaffolding is both (a) not specific to phonology and (b) false, since some writing systems are hieroglyphic. However, these claims are both self-contradictory and individually unwarranted. Scaffolding does not mean that *every* writing system ever invented is phonological. Rather, phonology appears to impose a constraint on the cultural evolution of writing systems, such that *mature* writing systems tend to encode phonological units (DeFrancis, 1989), and readers tend to automatically recover these phonological representations from print (for discussion, see Perfetti, 1985; Van Orden et al., 1990; Berent and Perfetti, 1995). Similarly, Everett’s first objection (“scaffolding is non-unique”) attacks a straw man. No one claims that scaffolding is a defining feature (i.e., a necessary and sufficient condition) of core knowledge, so Everett is right to note that these properties are also found in systems that are clearly invented by culture (“Burrito-making has its own unique features,” p. 8). However, while Everett may admire the unique design of burritos, enthusiasm for this observation is tempered by the fact that, unlike phonology, burrito design is neither universal, early nor spontaneously emerging. The conjunction of these features is suggestive of core knowledge.

## Conclusion

Everett is certainly right to question whether phonology is a system of core knowledge. I applaud his efforts to unveil the origins of language and his many contributions to the field. Summarizing the state of research in *The Phonological Mind*, I concluded, “While the core phonology proposal seems to presently offer the best explanation for the wide range of evidence considered in this book, the available evidence is insufficient to fully evaluate this hypothesis,” and I proceeded to indicate a number of open questions for future research (Berent, 2013a, p. 312). Resolution of these issues will require a nuanced theoretical analysis followed by careful experimental scrutiny. Inflammatory statements only hinder the progress of this enterprise. As Everett puts it, “… a spurious observation of a few phonologists is not likely to serve as an instinct” (Everett, 2016b, p. 6). Neither, by the same token, could such remarks possibly refute this hypothesis.

## Author Contributions

The author confirms being the sole contributor of this work and approved it for publication.

## Conflict of Interest Statement

The author declares that the research was conducted in the absence of any commercial or financial relationships that could be construed as a potential conflict of interest.

## Footnotes

1 All page citations of Everett (2016a) are based on a manuscript from August 03, 2015.
